# Toward a Global Bioethics: Principlism and the Problem of Political Legitimacy

**DOI:** 10.1111/bioe.13434

**Published:** 2025-06-08

**Authors:** Marco Annoni

**Affiliations:** ^1^ National Research Council of Italy (CNR) Interdepartmental Center for Research Ethics and Integrity (CID‐Ethics) Rome Italy

**Keywords:** global bioethics, medicine, morality, policy, principlism

## Abstract

Tom Beauchamp and James Childress's *Principles of Biomedical Ethics* introduced principlism—or the “four principles approach”—which has since become one of the most influential frameworks in contemporary bioethics. However, its potential to serve as a foundation for shared transcultural bioethical norms has elicited both substantial support and considerable critique. In this article, I analyze two notable attempts that utilize, or appear to be modeled after, principlism as a basis for global bioethics: Beauchamp and Childress's original formulation and the recently revised *International Code of Medical Ethics* by the World Medical Association. I argue that each model fails, but for different reasons. Beauchamp and Childress's account is rooted in particular moralities, making it suitable for guiding action in specific clinical contexts but ill‐equipped to handle global ethical pluralism. Conversely, the WMA's approach is deficient due to its undefined moral foundation and lack of political legitimacy. To address these shortcomings, I outline a third approach designed to make explicit the connection between principlism, global bioethics, and the problem of political legitimacy.

## 1 Introduction: Principlism, Moral Relativism, and Global Bioethics

In her book *Against Relativism: Cultural Diversity and the Search for Ethical Universals in Medicine*, bioethicist Ruth Macklin noted, “A long‐standing debate surrounds the question of whether ethics are relative to time and place. One side argues that there is no obvious source of a universal morality and that ethical rightness and wrongness are products of their cultural and historical settings. Opponents claim that even if a universal set of ethical norms has not yet been articulated and agreed upon, ethical relativism is a pernicious doctrine that must be rejected” [1].

Recently, this classical debate has been reignited because of a series of converging factors. First, global phenomena such as COVID‐19 and other pandemics, climate change, and new technologies like gene editing and artificial intelligence have had a worldwide impact, demanding global solutions. Addressing these challenges requires a coordinated approach across borders, cultures, and political communities. Second, globalization has increased interactions between health professionals and patients from diverse cultural, religious, and social backgrounds. In clinical settings where moral pluralism is highly valued, there is a growing need for new ethical norms that account for individual and sociocultural differences. Additionally, as medicine and clinical research become increasingly global, there is a pressing need to establish common ethical standards that apply regardless of socio‐cultural and geographical differences. Together, these factors raise the question of whether it is possible to identify a set of globally shared ethical norms despite our differences.

Some believe that principlism can provide a framework of norms that can be agreed upon on a global scale—that is, a basis for global bioethics. Developed over more than 40 years by Tom L. Beauchamp and James C. Childress in their classical *Principles of Biomedical Ethics* (henceforth, PBE), principlism—or “the four principles approach”—is one of the most influential theories of contemporary bioethics [2, p. 398]. As such, it has attracted its share of supporters and critics, especially regarding its ability to provide a non‐foundational transcultural set of bioethical norms.

Scholars have generally defended three positions on whether principlism is a suitable framework for global bioethics. Some, like Beauchamp and Childress, have argued that principlism is already up to the task. Indeed, the concluding paragraph of the PBE 8th edition reads, “Our theory of a framework of principles is committed to global bioethics by presenting universally binding norms that constitute ineliminable starting points for determining what is ethically acceptable in all societies. This theory rejects the hypothesis that morality is ultimately reducible to local, customary, or cultural rules” [2, p. 398]. On this view, principlism already provides a suitable theoretical basis for global bioethics.

Others disagree. Søren Holm, for instance, has argued that because the theory of the PBE “is developed from American common morality (and in reality only from a subset of that morality) it will mirror certain aspects of American society, and may, for this reason alone, be untransferable to other contexts and other societies” [3]. Similarly, other scholars have claimed that principlism embodies a fundamentally Western, white, and male‐centric view of morality—one that is reflected, for instance, in its focus on local decisional autonomy and individual informed consent [4]. In this view, principlism is structurally unfit for global bioethics, as it reflects only part of a much richer moral world.

Finally, others have maintained that principlism can provide a starting point for global bioethics, but only if we add important qualifications to this claim. In this article, I will defend a version of the third approach, arguing that principlism may provide a basis for global bioethics, but only if we understand it in a very “weak sense,” that is, as a minimal, content‐thin, procedural theory that lends itself to a rich set of different, yet equally legitimate, cultural specifications.

## 2 Principlism and Global Bioethics: An Impossible Trade‐Off?

Which account of principlism is best suited for global bioethics? At first, this might seem a trivial question: this account is simply the one outlined in the last edition of the PBE. This answer, however, would miss the mark in some important ways, and it would do so for reasons that follow directly from the theoretical account outlined by Beauchamp and Childress in the PBE.

To see why, it is necessary to clarify how, according to the PBE, any useful account of principlism is necessarily the co‐production of the universal norms of common morality and the content supplied by particular moralities. According to the PBE, “Common morality” is the “set of universal norms shared by all persons committed to morality. This morality is not merely a morality, in contrast to other moralities. It is applicable to all persons in all places, and we appropriately judge all human conduct by its standards” [2, p. 3]. By contrast, “particular moralities” include “the many responsibilities, aspirations, ideals, sentiments, attitudes, and sensitivities found in diverse cultural traditions, religious traditions, professional practice, and institutional guides” [2, p. 4]. The body of legal and interpretative norms in the Talmudic tradition, the Islamic reliance on Shari'ah‐based principles, and deontological and professional guidelines are all instances of “particular moralities” [2, p. 5].

While the norms of particular moralities have various sources, the universal norms of common morality are all identified in the same way through “considered judgments.” Considered judgments are “our most thoroughly examined moral beliefs,” the ones that throughout history have surfaced as useful shards through which we interpret and frame our moral life [2, p. 6]. The four mid‐level clusters of *prima facie* principles that define principlism (respect for autonomy, nonmaleficence, beneficence, and justice) all stem from these almost intuitionist and pre‐theoretical judgments, as do other norms of common morality such as universal virtues, moral ideals, and basic human rights. However, while considered judgments may guide us in identifying a cluster of relevant moral norms through consensus, it alone cannot provide such norms with the content needed to guide our actions in practice.

To this end, all norms of common morality must be specified. “Specification” is the process of “reducing the indeterminacy of abstract norms and generating rules with action‐guiding content” [2, p. 17]. As Henry Richardson has notoriously put it, specification narrows down the scope of general norms by spelling out where, when, why, how, by what means, to whom, or by whom the action is to be done or avoided [2, p. 18]. Importantly, specification occurs in two ways. First, a norm can be specified to accommodate the features of a particular case. For instance, the norm “do not harm” may be specified in light of a particular patient or clinical condition or the features of a new technology or clinical procedure. Second, a norm can be specified by integrating clusters of concepts, values, and norms from particular moralities. For instance, the norm “do not harm” may be specified in light of a particular religious and cultural view. And, indeed, as many have noted, also basic concepts like “autonomy,” “harm,” “reason,” or “justice” admit different and conflictual specifications.

Two important implications follow from this view. First, to be useful in practice, each principlist account must be, to some extent, specified. Crucially, both the universal, content‐thin norms of common morality and the particular, content‐rich, and culturally situated norms of particular moralities are always required to guide our actions. Paraphrasing Kant's saying, without a reference to common morality, our moral norms are blind; without specification, they are empty [5].

Second, the same abstract cluster of norms may be legitimately specified in different ways: what matters is only that each specification does not conflict with common morality. Hence, two specifications at the same level of abstraction—but that have been refined through different culturally situated moralities, empirical experiences, particular cases, and deliberative processes—may eventually conflict with each other and yet be both compatible with common morality.

**Figure 1:**
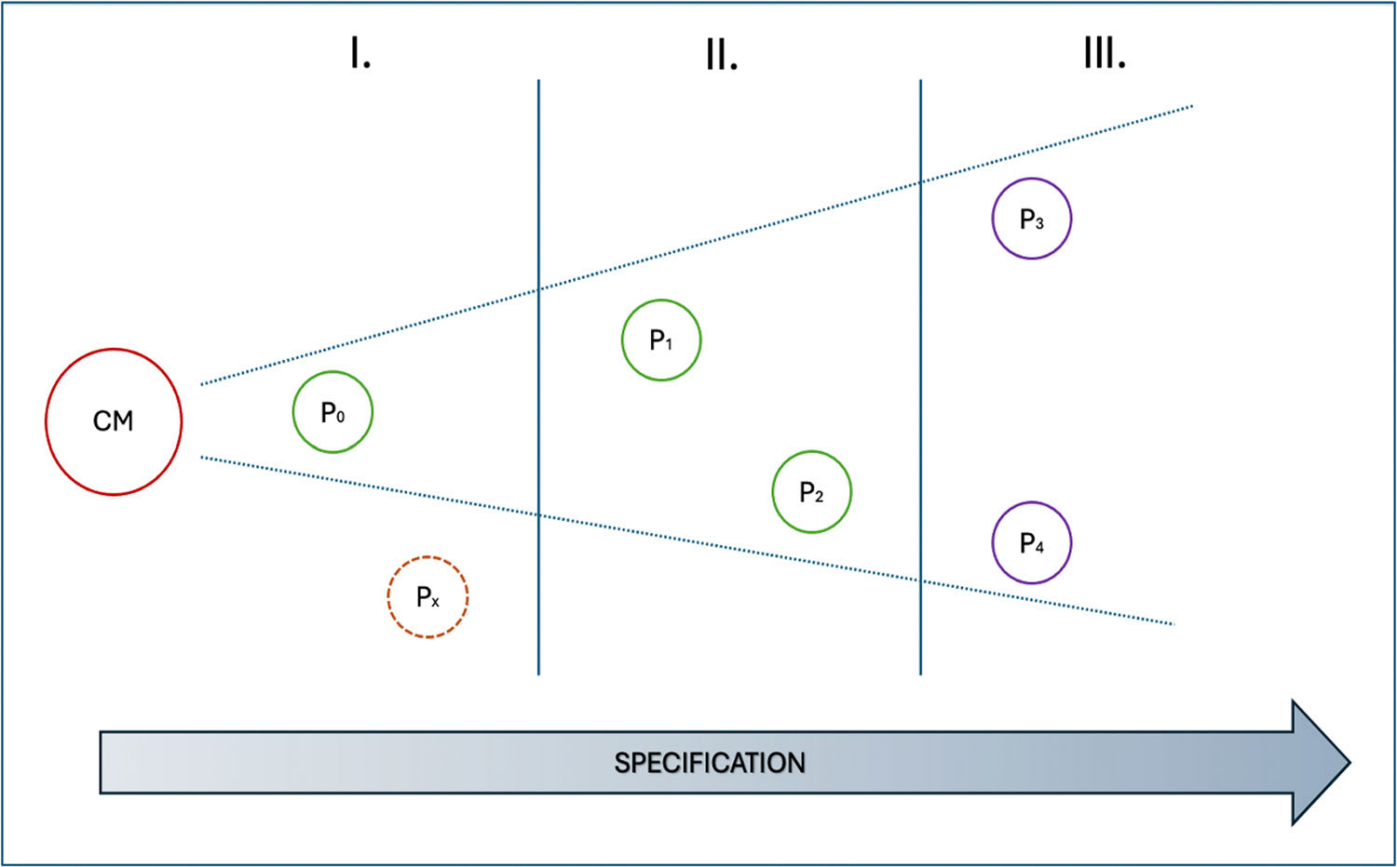
Gradient of specification from universal common morality to particular moralities.

If this is correct, then it follows that there is not just one right principlist account, but many, and that we can also place them on a continuum scale depending on how much they have been specified in light of particular moralities, as shown in Figure 1.

The least specified accounts (P0, P1, P2) will be closer to the universal norms of common morality, while the more specified ones (P4, P5) will be farther away. No account is likely to be completely universal or specified. Also, at each gradient of specification, more than one coherent account is possible (P4, P5) depending on which particular morality and facts have been used to specify it.

Identifying where a particular account falls along this continuum is difficult. Nonetheless, we can distinguish three general cases. The first (I) includes all accounts so unspecified that virtually anyone would acknowledge them as very close to common morality (P0). These accounts have the highest chances of providing a good basis for global bioethics, but they are also the least useful in practice, as they are very content‐thin. The second (II) includes all accounts sufficiently specified to guide actions in most cases, and that most could endorse as compatible with their particular moralities (P1, P2). Finally, the third (III) category includes all accounts heavily specified (P3, P4). These accounts are so rich in content that they can effectively guide actions in most real‐world cases; at the same time, they are so specified that people endorsing other particular moralities might legitimately reject them as incompatible with, or not representative of, their worldview.

To which category does the PBE account belong? Those who believe that this account already provides a strong foundation for global bioethics would likely place it in the first category. However, this claim is highly questionable. The latest edition of the PBE spans nearly five hundred pages and offers a rich, highly detailed account of principlism, refined over forty years of engagement with numerous exemplar cases and a robust scholarly debate. It is thus hard to deny that the 8th edition PBE account of principlism is already highly specified. Importantly, it cannot be any other way: as the primary goal of the theory in the PBE is to aid doctors in deciding in real clinical scenarios, a high level of specification is not only expected but also demanded of such an account to be of any utility.^1^

To be clear, my claim here is not that we should accept or reject Beauchamp and Childress' PBE account of the moral foundations of principlism, nor that we should accept or reject their distinct specification of the system. Rather, my claim is only that, *if* one accepts their account of the relationship between common morality and particular moralities via specification, *then* one inevitably faces an insoluble trade‐off between practical utility and global applicability. Either their account is sufficiently specified to guide doctors' actions in real scenarios, or it is sufficiently unspecified to provide a suitable basis for global bioethics. In short, if one aims at guiding health professionals' decisions in most real‐world clinical scenarios in a specific cultural setting, then one cannot also have the cake of global bioethics and eat it too—at least not within the principlist framework espoused in the PBE.

One possible strategy to resolve this conundrum is to relinquish a higher level of specification and adopt a relatively unspecified version of principlism—one that remains sufficiently general to accommodate its various potential coherent specifications. However, pursuing such an approach raises two key issues. The first concerns how to define this minimal theoretical account. The second issue concerns how this principlist framework can be specified and accepted as legitimate by all those engaged in the global bioethics discourse. Addressing this latter issue is crucial, as it entails navigating complex theoretical and practical challenges frequently overlooked. These challenges are explored in the following section, where I critically analyze the recent attempt by the World Medical Association to crystallize a “global ethos of medicine” through its revised *International Code of Medical Ethics*.

## 3 The WMA International Code of Medical Ethics and the Global Ethos of Medicine

The World Medical Association (WMA) was established in 1947 to promote the highest ethical standards for physicians around the globe. Today, it represents over ten million doctors and is one of the most influential sources of ethical codes and deontological guidelines for doctors. Its key documents include the *Declaration of Geneva* (a modern version of the Hippocratic Oath), the *Declaration of Helsinki* (outlining fundamental ethical principles for clinical research involving human subjects), and the *International Code of Medical Ethics* (ICoME), which sets common ethical principles for physicians in their relationships with patients, colleagues, and society.

In 2022, the WMA approved a revised version of the ICoME noteworthy for three reasons. First, the revised code resulted from a 4‐year deliberative process involving multiple confrontations with the WMA's international representatives as well as with the global community of bioethics experts and civil society. This process culminated in the unanimous adoption of the revised code during the WMA 73rd Annual General Assembly. In the words of the members of the WMA drafting group, the new ICoME was born as “an ethical document, adopted in a political procedure” [6].

Second, the revised ICoME significantly expands and redesigns doctors' ethical duties. Besides some expected updates, the 2022 ICoME also introduces some substantial and a few potentially controversial novelties. For instance, it introduces for physicians a duty to “practise medicine in ways that are environmentally sustainable” (art. 10), to “attend to their own health, well‐being, and abilities” (art 28), and to “indicate if their own opinions are contrary to evidence‐based scientific information” (art. 35). It also limits conscientious objection to those situations in which “an individual patient is not harmed or discriminated against” and her health is not endangered (art. 29) [7].

Crucially, the revised code now stresses that physicians must provide care with the utmost respect not only for human life and dignity but also “for the autonomy and rights of the patient.” Accordingly, the 2022 ICoME features a new part (art.15‐18) on the importance of securing patients' *individual informed consent* and respecting their (local decisional) autonomy. The duty to respect the autonomy of each individual patient is thus extended globally, becoming a universal ethical obligation regardless of the social, political, or anthropological context in which physicians operate.

The significance of these changes, which received unanimous approval from all WMA representatives—including the delegate from the Vatican—should not be underestimated. It illustrates that, under certain conditions, it is possible to reach consensus on a set of shared ethical norms for medical practice, even among stakeholders with markedly diverse sociocultural backgrounds. While this conclusion is contingent on various caveats and the fulfillment of key prerequisites, as will be elaborated below, it bears considerable practical importance. Contrary to the assumption that moral, cultural, and political differences are too vast to permit meaningful normative consensus in a contentious domain such as medicine, the global adoption of the 2022 ICoME offers a compelling and concrete counterexample. The fact that over ten million physicians worldwide, along with the billions of patients they serve, can now refer to a unified code of conduct—transcending differences in culture, religion, values, and sociopolitical contexts—is an unprecedented development in the history of medicine and bioethics, and one that warrants serious consideration.

Third, the new ICoME was elaborated with the explicit aim of being *“*applicable globally — to different cultures, denominations—religious and secular—and political environments” [6, p. 163], that is, to crystallize “the global ethos” of medicine. As explained again by the chair of the working group in dedicated articles as well as in a thematic panel during the 2024 World Congress of Bioethics, this revision was required, among other things, to reassure the world's population “of the agreed core moral commitments of the world's doctors. The moral answer to globalization is a global ethos. The ICoME is a document with the claim to such a global ethos in the limited context of medical ethics” [6, p. 163]. Thus, according to the WMA, the revised ICoME provides the closest approximation to a minimal core of globally agreed upon ethical norms for the medical profession. As such, it represents a fascinating case study for the debate over the possibility of a global bioethics.

Interestingly, the revised ICoME also has a deep yet ambiguous relationship with principlism. On the surface, the 2022 ICoME is a deontological document uncommitted to any specific moral theory. Moreover, according to Parsa‐Parsi and colleagues, there are two plausible arguments supporting the claim that the WMA code is specifically *not* based on principlism. One is simply that the ICoME “does not claim to be based on any moral theory” [6, p. 168]. However, without further justification this claim is, well, just a claim—and not a plausible, let alone persuasive, argument.

The second argument is that the ICoME recognizes two more fundamental principles—“respect for human life” and “respect for human dignity”—besides the four classical ones. While true, this only proves that the ICoME account is not reducible to the specific account outlined in the PBE, not that the framework of the revised code is not based on, or at least compatible with, a more general principlist account. As explained in the previous section, the set of norms in the ICoME may represent just another possible specification of common morality—one that is equally legitimate but different from the one in the PBE. This other account may (or not) include these two principles as specifications of the other four, or as stand‐alone additional principles alongside the classical four.

In fact, at a closer look, the WMA code appears not only compatible but also somehow modeled after a principlist account – or, at least, heavily influenced by it. To understand this, it is essential to look beyond the final ICoME text and instead focus on the articles written by the WMA members involved in its revision.

In an essay on the genealogy and scope of the revised code, the members of the drafting groups explain that the section headed “general principles” in the 2022 ICoME “can be seen to comprise basic ethical principles; specifications of such principles; required virtues and virtuous behaviours; and specific deontological obligations/rules/duties. This range of ethical content in the ICoME […] echoes ‘the lived experience' of doctors internationally and represents the considered common morality of the profession” [6, p. 166]. Indeed, these references to “common morality,” “principles,” “specification,” “considered morality” (i.e., doctors' “considered judgments”), and to a normative framework essentially comprising duties and virtues are largely compatible with—if not directly derived from—a standard principlist account, including the one outlined in the PBE.

Furthermore, while the four PBE principles are not explicitly mentioned in the ICoME, they are nonetheless “incorporated within the approach to medical ethics taken by the WMA,” for they are “helpful for organising and explaining the very many more specific principles in the Code and the various specified virtues” [6, p. 167]. Thus, most of the norms in the revised ICoME can be framed around the four principles, and doing so can also help explain—that is, specify—their content. More generally: “the four principles approach, used as an ‘approach’ to medical ethics rather than as a basic moral theory, and added to (as in the ICoME), rather than replacing whatever overarching moral theory a doctor adheres to, can provide a set of prima facie moral commitments that all doctors can share. The ICoME is also compatible with claims that these principles could provide important basic elements of an international and intercultural moral language and even of a basic moral analytic framework that can be shared with colleagues and patients, regardless of the wide variety of ‘overall’ moral perspectives that those colleagues and patients themselves might adhere to in our increasingly globalised world” [6, p. 167].

This passage is noteworthy for two reasons. First, it misrepresents principlism. Even in the PBE, the four principles are not presented as a “basic moral theory.” Rather, they are clusters of norms meant to provide a specification of the aspects of common morality most relevant to biomedicine. A key feature of principlism is precisely that of rejecting a foundational approach in favor of an agreement on a minimal set of mid‐level *prima facie* principles. As shown by the quote above, this is more or less the meta‐ethical approach beneath the 2022 ICoME, and this approach *is* compatible with being based on a “principlist account,” however implicit that might be in the final text. The claim that the ICoME is not based on *any* moral theory, thus, is better interpreted as meaning that while the ICoME is not based on any foundational moral theory (deontology, consequentialism, virtue ethics, religious revelation, etc.), it adopts—or is at least compatible with—a procedural account of bioethics based on a minimal set of mid‐level duties and agreed‐upon focal virtues.

Second, not only can most of the norms in the ICoME be viewed as specifications of the four principles but also these norms should, accordingly, be understood as *prima facie* duties—that is, as binding moral obligations to be followed unless there is a sufficiently strong justification to act otherwise. According to Beauchamp and Childress, and following D.W. Ross's account, this entails that when *prima facie* duties conflict, doctors should specify each norm and balance the reasons supporting each alternative course of action. This process helps determine their “actual duty” in that particular case: what should be done all things considered. This is clearly another procedural hallmark of principlism.

Thus, even though the ICoME does not explicitly reference principlism, it effectively incorporates it, both at the level of its fundamental moral elements and at the meta‐ethical level of how those norms or duties should be interpreted and applied—that is, as *prima facie* duties. This is relevant in the light of the 2022 ICoME practical importance, for it means that such an agreement was reached by adopting many features typical of a procedural and principlist approach to bioethics—another fact that should not be downplayed within the current debate on global bioethics.

However, despite its merits, the 2022 ICoME also has a few crucial limitations, especially with respect to its pretense of having successfully crystallized the global ethos of medicine—that is, of being a good basis for global bioethics. These limitations are essentially three: conceptual vagueness, lack of an explicit procedure to solve internal conflicts between its principles, and lack of adequate political legitimacy. As I have already analyzed these limitations in depth elsewhere, here, I will focus only on the crucial one for the scope of the present article: the ICoME lack of political legitimacy [8].

The core of the critique lies in the observation that, although the 2022 ICoME positions itself as a global standard for clinical practice, its development and ratification were ultimately carried out by a single stakeholder group: physicians (and not even all of them, as the WMA neither encompasses all practicing doctors nor other healthcare professionals). This process results in a clear power asymmetry: while the ICoME will impact a wide range of actors—directly affecting physicians and indirectly shaping the roles of other healthcare workers and patients—only a limited group had the authority to determine which norms were adopted and how they were formulated. Admittedly, as previously noted, the WMA incorporated several instances of public consultation during the code's revision. However, these brief and sporadic consultations are insufficient to substantiate the legitimacy of extending the norms adopted by the WMA assembly to all relevant parties.

To draw a parallel, imagine if the wealthiest countries in the world decided to establish a set of common ethical norms to address global bioethical issues. In formulating these norms, they might organize several conferences and issue open calls for feedback, providing other countries the opportunity to offer their perspectives. However, if only the wealthiest countries retain the decision‐making power—deciding which norms are considered, which feedback is incorporated or disregarded, and ultimately voting on the adoption of the final norms—it would be reasonable for other countries to reject the binding nature of these norms on the grounds that they were not given a genuine opportunity to participate in their development.

Robert Veatch, in an influential article in 1979, had already warned about the limits of overextending the normative validity of deontological codes for the medical profession [9]. According to Veatch, there is a substantial difference in terms of legitimacy between the norms concerning “the ethics of a profession”—equivalent to internal deontology, or “norms or courtesy” among colleagues—and the ethical norms that must be respected in the exercise of a professional role. While the norms within a profession concern only the members of that community, the norms related to the exercise of a professional role, such as that of a physician, also concern patients and society. For this reason, while the first set of norms can be justified by an internal political agreement among peers, “another source of role‐specific duties for professionals must exist if those duties justify interactions outside the group” [9, p. 13]. This alternative source may be of three kinds: a shared metaphysics (as with religious groups and communities), a foundational theory (as with deontology or consequentialism), or a political agreement between all those that will be significantly impacted by such norms.

As the ICoME (and principlism) rejects both theoretical and metaphysical foundationalism, its legitimacy may only come from political agreement. But this agreement, to be considered legitimate, must include—in a way of the other—anyone who will be affected by these norms. Otherwise, “If professionals have agreed among themselves that the physician's duty is to do what he thinks will benefit the patient or to do no harm to the patient or even to do what professional groups believe is in the public interest, it still must remain an open question whether society concurs that such a principle is reasonable for governing relationships with members of the society” [9, p. 15]. Following Rawls, Veatch then argues in favor of a tripartite “social contract”: one between all members of society, one between doctors and patients, and one between each individual doctor and each individual patient.

Veatch's proposal remains thought‐provoking and continues to warrant further examination. However, for the purposes of this article, it suffices to acknowledge his central argument: that a set of ethical norms governing a role‐based profession such as medicine can only be considered legitimate if all those significantly impacted by these norms are given a fair opportunity to participate in, or have their perspectives represented during, the processes of their formulation and adoption. Following this reasoning, a global set of anti‐foundational bioethical norms cannot be genuinely legitimate without a corresponding global political process, one that ensures equitable representation and participation for all affected stakeholders.

In short, viewed solely as “a deontological document listing various ethical guidelines drafted by a profession for its professionals” [6, p. 168], the new ICoME represents a significant achievement, not only for the WMA but also for all patients who will interact with the millions of its members. That such an agreement was reached is remarkable, as it is the recognition that to achieve this result, many of the theoretical and practical hallmarks of principlism have indeed been instrumental. However, despite its claims of global representativeness, and a well‐intended and inclusive review process, the WMA's revised ICoME cannot provide a good basis for global biomedical ethics. Being only a code of self‐regulation developed and ratified exclusively by WMA members—a professional society rather than a public institution—it lacks the adequate political legitimacy to be accepted as representative, and therefore binding, also by others who will nonetheless be affected by such norms.

## 4 Conclusions: Principlism, Global Bioethics, and Political Legitimacy

My examination of these two attempts to use principlism as a basis for global bioethics shows both to be problematic. Beauchamp and Childress's PBE account fails because it is already too specified, and thus, some might legitimately claim that it cannot represent, or is in conflict with, their particular moralities. The WMA's attempt fails because its revised ICoME lacks a more content‐rich theoretical basis, an explicit procedure to solve internal conflicts, and adequate political legitimacy.

In between these extremes is a third possible view, which I shall provisionally define as “weak principled intuitionism” or “weak principlism” for lack of a better term. A detailed account and defense of this account exceed the purpose and limits of this article. Nonetheless, even a rough outline is useful to make explicit the direct and often overlooked link between principlism and the problem of political legitimacy.

So, how would such an account be structured to provide a good basis for global bioethics? Clearly, on the one hand, it would need to be much weaker—that is, less specified—than Beauchamp and Childress' PBE account to leave room for most, if not all, possible coherent specifications. At the same time, it would need to be stronger than the WMA one, for it will require an explicit albeit minimal theoretical framework and a procedure to interpret and manage common moral issues and dilemmas. As a principlist account, it would also need to be committed at least to the following ideas:

i. Common moral issues are interpretable in terms of a conflict between mid‐level *prima facie* moral rules that ought not to be violated without adequate justification or between moral ideals (e.g., virtues, character traits) that ought to be generally encouraged and promoted;
ii. Interpreting, managing, and solving common moral issues require specification and balancing. Specification is needed to guide the identification and agreement of the initial set of moral rules and moral ideals, as well as to provide guidance for action in light of particular cases, concepts, phenomena, and experiences. Balancing is needed to overcome moral dilemmas by identifying which, among all conflicting *prima facie* moral rules and ideals, ought to prevail in each particular case, all things considered.

A commitment to these basic ideas is required for any approach or moral system without mid‐level *prima facie* principles, as its basic elements could hardly be defined as an instance of “principlism.”

If this is correct, then, account would also possess other noteworthy features. First, such a framework would not stipulate a fixed number of foundational moral rules or ideals, as there is no predetermined method for establishing these independently of an agreement among the relevant parties. Second, while this framework would be compatible with the PBE's theory of common morality, it would not require it; the participants could agree on a set of norms without consensus on the underlying sources of their moral agreement (or disagreement). Similarly, it would allow for other elements of PBE principlism—such as its specific account of “moral status” or its identification of five “focal virtues”—without mandating their inclusion. Third, it would remain an open question whether and to what extent a system derived from such a weak principlist framework could be applied to issues beyond those that initially motivated its creation. For instance, distinct communities might converge on shared norms to address urgent, collective problems (e.g., a pandemic) while still maintaining divergent value systems on unrelated matters (e.g., secular vs. religious worldviews). The applicability would ultimately hinge on the nature and content of the principles and ideals agreed upon.

Fourth, even in its this minimal form, to operate any principlist account, one must address the following questions: Who holds the authority and power to decide? Who determines which moral rules and ideals are deemed fundamental? Who evaluates the content of “considered judgments” and whether consensus exists around them? Who decides how norms are specified in relation to particular facts and contextual guidelines, that is, particular moralities? When faced with equally legitimate but conflicting specifications, who has the authority to choose which one to prioritize and which to set aside? And, ultimately, who decides which principle should take precedence in cases of conflict?

Addressing these questions is essential for any principlist framework, strong or weak. Given that a central tenet of principlism is anti‐foundationalism, achieved through a shared agreement on mid‐level, *prima facie* norms, the challenge of legitimacy remains consistently relevant. Neither an appeal to a universal common morality, as articulated in the PBE, nor reliance on an internal self‐regulatory process, such as the one conducted by the WMA, can sufficiently resolve this issue if the objective is to formulate a genuinely global set of bioethical norms. In both instances, the resulting framework—no matter how well intentioned or meticulously constructed—remains vulnerable to the critique that it has not adequately considered the diverse perspectives and values of all stakeholders.

Thus, to be genuinely inclusive and legitimate, any principlist approach necessitates both a robust moral and political theory. Such a political theory must clarify who is vested with legitimate decision‐making authority and delineate the processes through which consensus is achieved among all relevant parties, particularly at critical procedural junctures. Without this comprehensive and fully explicit political underpinning, a principlist framework risks devolving into a construct that is validated solely by the agreement of those wielding power and authority at any given time.

This necessity for a complementary political framework extends to any attempt at establishing global bioethical standards and that renounce to moral foundationalism. It underscores the importance of designing inclusive procedures that allow for equitable representation and engagement of diverse stakeholders in the formulation of common bioethical norms. Without such measures, even the most rigorously developed and well‐intended ethical systems may fall short of achieving true legitimacy.

## Data Availability

Data sharing is not applicable to this article as no data sets were generated or analyzed during the current study.

## Endnotes

1 Perhaps, a fairer estimation would place the PBE account somewhere in between the second and third category. This would reflect the current state of the debate, in which Beauchamp and Childress's account is widely influential despite being highly specified, so that some may legitimately argue that it is incompatible with—or too far away from—their particular moralities.
